# Visible emission from bismuth-doped yttrium oxide thin films for lighting and display applications

**DOI:** 10.1038/s41598-017-17567-9

**Published:** 2017-12-11

**Authors:** Adriana Scarangella, Filippo Fabbri, Riccardo Reitano, Francesca Rossi, Francesco Priolo, Maria Miritello

**Affiliations:** 1 CNR IMM-MATIS, Via S. Sofia 64, 95123 Catania, Italy; 2 Center for Nanotechnology Innovation @NEST, Istituto Italiano di Tecnologia, Piazza San Silvestro 12, 56127 Pisa, Italy; 3 IMEM-CNR, Parco Area delle Scienze 37a, 43124 Parma, Italy; 4 Dipartimento di Fisica e Astronomia, Università di Catania, Via S. Sofia 64, 95123 Catania, Italy; 5 Scuola Superiore di Catania, Università di Catania, Via Valdisavoia 9, 95123 Catania, Italy

## Abstract

Due to the great development of light sources for several applications from displays to lighting, great efforts are devoted to find stable and efficient visible emitting materials. Moreover, the requirement of Si compatibility could enlarge the range of applications inside microelectronic chips. In this scenario, we have studied the emission properties of bismuth doped yttrium oxide thin films grown on crystalline silicon. Under optical pumping at room temperature a stable and strong visible luminescence has been observed. In particular, by the involvement of Bi ions in the two available lattice sites, the emission can be tuned from violet to green by changing the excitation wavelength. Moreover, under electron beam at low accelerating voltages (3 keV) a blue emission with high efficiency and excellent stability has been recorded. The color is generated by the involvement of Bi ions in both the lattice sites. These peculiarities make this material interesting as a luminescent medium for applications in light emitting devices and field emission displays by opening new perspectives for the realization of silicon-technology compatible light sources operating at room temperature.

## Introduction

Luminescent thin films are gaining increasing attention as visible emitters for optoelectronic devices, bio imaging, drug delivery, solid state lasers as well as flat panel displays (FPDs)^1^. Among the display technologies, light emitting devices (LEDs) and field-emission displays (FEDs) have many advantages, such as low power consumption, good compactness, long lifetime, low weight to size ratio, high resolution, quick response, etc.^2^. However, being FEDs composed by an array of aligned field emitter tips cathodes opposite to a phosphor anode, their development has been limited by the complexity of the device fabrication and by the presence of contaminants released under the high-electron bombardment and related to the used phosphor. Therefore a big part of the scientific community working on FEDs is nowadays focused on the development of novel stable materials emitting in the visible range with high luminance, good chromaticity, long service time, high efficiency at low voltages (<5 keV)^2,3^. Similarly, for white or tunable LEDs application, many efforts are devoted to developing down-conversion luminescent materials for phosphor-converted LEDs (pc-LEDs) that are competitive in view of the low cost and the simplicity of the control circuit^4,5^. Different approaches have been already proposed by combining blue chips with yellow YAG phosphors or near UV (NUV) chips with separated red-green-blue (RGB) tricolor phosphors^4^. The better chromatic flexibility, higher stability and higher efficiency make NUV-based pc-LEDs expected to have great potential applications in the field of solid state lighting. Thus, nowadays a great interest is still generated in the development of new phosphors for blue, green, or red emission upon NUV excitation^2–7^.

In particular, the choice of activator ions and host materials are subject of investigation. For the first aspect, rare earth (RE) ions have been playing an important role due to the abundant emission colors in the whole visible range owing to their 4 f-4 f transitions, but unfortunately with very small excitation cross sections and thus low luminous efficiency and a poor controllability of emission colors. Thus REs ions exploiting 5d-4 f transitions, such as Eu^2+^ or Ce^3+^, have been preferred and recently also transition and post-transition metals have been proposed^8,9^, owing to their higher excitation cross section and peculiar optical properties. About the hosts, the most commercially available for FEDs are the sulfide-based ones, such as CaSO_4_:Tb, Na^10^, but unfortunately they suffer from some limits, such as the quick degradation due to the corrosive sulfide gases produced under high-electron bombardment^2^. To overcome these drawbacks, many alternative RE-doped nanomaterials^11,12^, and phosphors^3,13^, have been proposed, among whom several RE doped oxides, such as Y_2_SiO_5_:Ce^3+^ ^14^, LiAl_5_O_8_:Tb^15^ and ZnGaO_2_^16^ and nitrides, such as Eu:AlN^17^, have been studied as possible solution.

In this work, we investigate the optical properties of bismuth doped yttrium oxide thin film, proposing it as a suitable efficient luminescent material for silicon-compatible pc-LEDs and FEDs technologies. Indeed, yttrium oxide is a transparent material that is considered a promising host for down- and up-conversion^18–20^ and for the realization of reliable luminescent devices, due to its good chemical durability, thermal stability, and low phonon energy. In addition, yttrium oxide thin film has been proven to be a well-adapted host to incorporate high concentration, up to 10^22^/cm^3^, of optically active trivalent ions in Y substitutional position^20,21^. Moreover, both the developed complementary metal-oxide-semiconductor (C-MOS) compatible synthesis techniques used to realize good quality yttrium oxide thin films and its demonstrated high thermal stability on silicon^22^ make this host suitable for fabrication by using mainstream microelectronics large scale manufacture technique. Thus it could permit to enlarge the range of applications inside microelectronic chips. In particular bismuth has been demonstrated to be stabilized in Y_2_O_3_ in the optically active 3+ oxidation state^21,23,24^, that is characterized by permitted optical transitions from its ground state, 6 s^2^, to the first empty orbitals, thus resulting in a very high excitation cross section, up to 10^−17^ cm^2^ ^21,25^, and very short radiative lifetime (~10 ns)^24^. In addition, since the 6 s electrons are not shielded by outer shells, such as for REs, the intensities and the positions of the emission peaks are strongly dependent on the local field felt by the ions, and therefore by the embedding host. Efficient green-blue emission from Y_2_O_3_:Bi^3+^ powders has been recently demonstrated^26,27^, while very few works report the optical properties of thin films^28,29^. Indeed, phosphor thin films have been recently proposed to replace the conventional powder phosphors due to several advantages, such as the lower operation voltage and the endurance to much higher power densities without degradation both for LEDs and FEDs. In addition, although the emission intensities are limited by the light trapping inside the luminescent layers, phosphor thin films also offer other strengths, such as higher contrast and resolution, better thermal stability, superior thermal conductivity, a high degree of uniformity and better adhesion to the substrate compared with the conventional display screen prepared by the direct deposition of phosphor grains^2^. These advantages make possible to define smaller pixel spot size, which is very important particularly for microdisplays.

Herein in particular, we will show the Y_2_O_3_:Bi optical properties under optical and electrical pumping by evidencing the excitation mechanisms of Bi ions and the perspectives of this material for the application in tunable pc-LEDs and in FEDs.

## Results and Discussion

**Figure 1:**
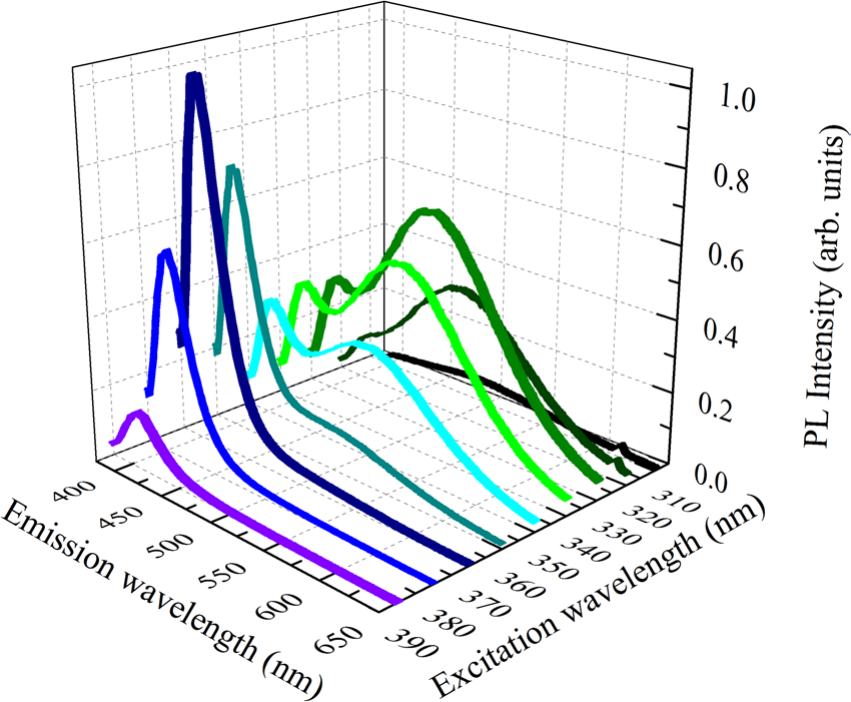
Photoluminescence from Y_2_O_3_:Bi under UV excitation. The RT PL spectra are reported for several excitation wavelengths between 300 nm and 400 nm.

In order to study the optical properties of dissolved Bi ions in Y_2_O_3_ host, PL spectra have been firstly recorded by scanning the excitation wavelength, λ_exc_, in the ultraviolet range between 300 nm and 400 nm that potentially can involve all the possible Bi oxidation states. Under these excitation conditions, no PL emission has been observed from the undoped thin film, since Y does not exploit radiative transitions. On the other hand, from the Y_2_O_3_:Bi thin film an intense PL emission has been detected in the visible range for all the excitation wavelengths, as shown in Fig. 1. By looking more deeply, the PL shape appears strongly dependent on the excitation wavelength. In particular, under λ_exc_ between 370 nm and 400 nm the PL emission is characterized by a peak in the blue region, centered at about 406 nm. By further decreasing λ_exc_ the peak intensity at 406 nm decreases, while a further contribution appears in the green region, centered at about 500 nm, whose intensity is increasing up to 330 nm excitation wavelength.

**Figure 2:**
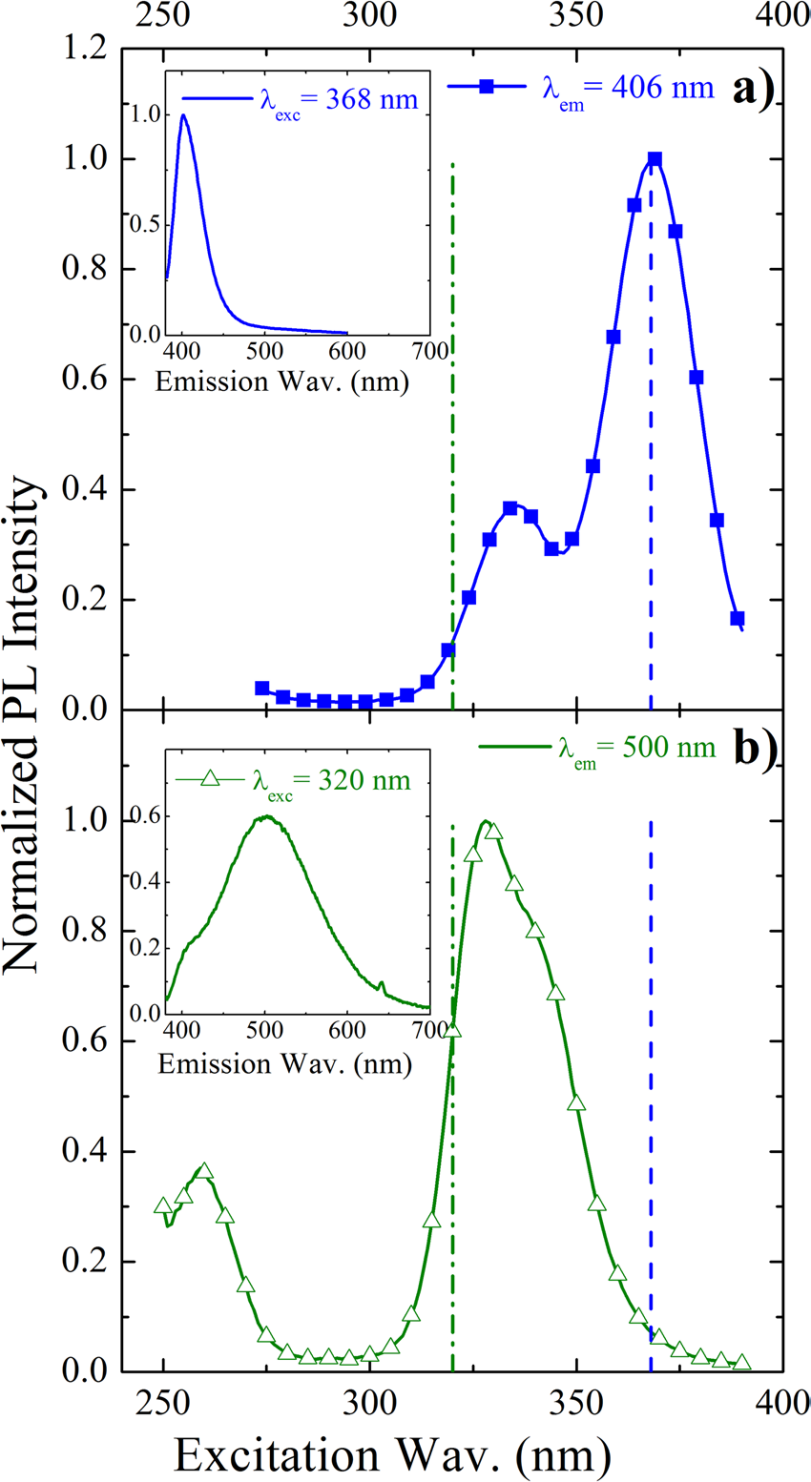
Excitation and emission spectra of two Bi sites. RT normalized PLE spectra from Y_2_O_3_:Bi recorded at two different emission wavelengths: (**a**) at 406 nm, related to Bi^3+^ ions in C_2_ sites and (**b**) at 500 nm, related to Bi^3+^ ions in S_6_ sites. The relative emission bands of Bi under (**a**) 368 excitation wavelength and (**b**) 320 nm excitation wavelength are also shown as insets. The two chosen excitation wavelengths for the inserts are also indicated in the main panels as dashed lines.

To elucidate the origin of these two features, photoluminescence excitation (PLE) spectra have been recorded at 406 nm, reported in Fig. 2(a) as line and closed squares, and at 500 nm, in Fig. 2(b) as line and open triangles.

The two excitation spectra appear totally different. In particular, for the blue emission the excitation spectrum is composed by the convolution of two peaks at about 334 nm and 368 nm (Fig. 2(a)), while for the green emission the correspondent excitation band seems to be composed by an asymmetric peak at around 330 nm and a further peak at about 258 nm (Fig. 2(b)). The two excitation bands around 350 nm can be attributed to the same transition of Bi^3+^ ions, ^1^S_0_ → ^3^P_1_, where the first excited energy level, ^3^P_1_, splits differently by depending on the site occupied by Bi^3+^ ion. Indeed, in our Bi-doped samples, Bi ions are well dispersed in the body centered cubic Y_2_O_3_ lattice in substitutional Y positions, as demonstrated by the unchanged XRD spectrum with respect to the undoped sample in ref.^30^, therefore Bi^3+^ ion can occupy two cation sites having different symmetries, named C_2_ (non-centrosymmetric, monoclinic symmetry) and S_6_ (centrosymmetric, rhombohedral symmetry)^23,24^. The sites are characterized by a different redistribution of the oxygen vacancies, in particular along the diagonal of the Y_2_O_3_ cubic cell for the S_6_ site and along one of the face diagonals for the C_2_ site. These different chemical surroundings induce strong variation in the crystalline field felt by the cation, by generating the different spectroscopic properties. In particular, when the emission wavelength is fixed at 406 nm in Fig. 2(a) the two excitation peaks at 334 nm and 368 nm can be associated to the ^1^S_0_ → ^3^P_1_ transition, where the ^3^P_1_ excited state splits in the doublet [^3^A_u_, ^3^E_u_] typical of Bi^3+^ in the S_6_ site^23^. Therefore under 368 nm, corresponding to the ^1^S_0_ → ^3^P_1_(^3^E_u_) transition of Bi^3+^ in S_6_ site, the sharp blue emission at 406 nm, FHWM of about 40 nm, in the insert of the same Fig. 2(a), is maximized. This blue emission, associated to the ^3^P_1_(^3^E_u_) → ^1^S_0_ transition, is Stokes shifted^23,24^, with respect to the relative excitation band, as expected for the post-transition metals with ns^2^ electronic configuration. The Stokes shift was calculated to be 2543.36 cm^−1^ for the S_6_ site.

When the emission wavelength is fixed at 500 nm, the broad and asymmetric excitation band peaked at 330 nm in Fig. 2(b) can be ascribed to the same transition ^1^S_0_ → ^3^P_1_, where ^3^P_1_ splits in the triplet [^3^A, ^3^B, ^3^B] owing to the presence of the crystalline field and the occurrence of spin-orbit coupling when Bi^3+^ is in the C_2_ site. The additional excitation peak in the deep UV, at 258 nm, can be associated to the transition from ^1^S_0_ to the higher energy level ^1^P_1_, totally permitted by the selection rules^24^. Thus, when the sample is excited under 320 nm excitation, that is in resonance only with the excitation band of Bi^3+^ in the C_2_ site and out of resonance with the one of Bi^3+^ in the S_6_ site, the PL emission is characterized by a main broad peak at about 500 nm and with FHWM of about 135 nm, in the insert of Fig. 2(b). It can be associated to the convolution of the Bi^3+ 3^P_1_ (^3^A, ^3^B, ^3^B) → ^1^S_0_ (^1^A) transitions of Bi^3+^ ions in the C_2_ site. In this case, the broader PL shape and the larger Stoke shift (10120.48 cm^−1^) are due to the stronger asymmetric nature of the C_2_ site. Anyway, the Stokes shifts calculated for the S_6_ and C_2_ are compatible with those reported for the Y_2_O_3_:Bi^3+^ bulk powders prepared by conventional solid-state methods (2500 and 10000 cm^−1^ for the S_6_ and C_2_, respectively^24^).

In conclusion, the origin of the blue and green emissions is clearly associable to the same ^1^S_0_ → ^3^P_1_ Bi^3+^ transition respectively when Bi^3+^ ion is in the S_6_ and C_2_ site. Moreover, they can be alternatively switched on (or off) by properly varying the excitation wavelength since the respective excitation bands are maximized in different spectral regions. In the narrow region of overlap, between 345 and 355 nm excitation, the two contributions reach equal intensities by determining a broad PL emission ranging from 400 nm to 650 nm with a whole FWHM of 183 nm.

**Figure 3:**
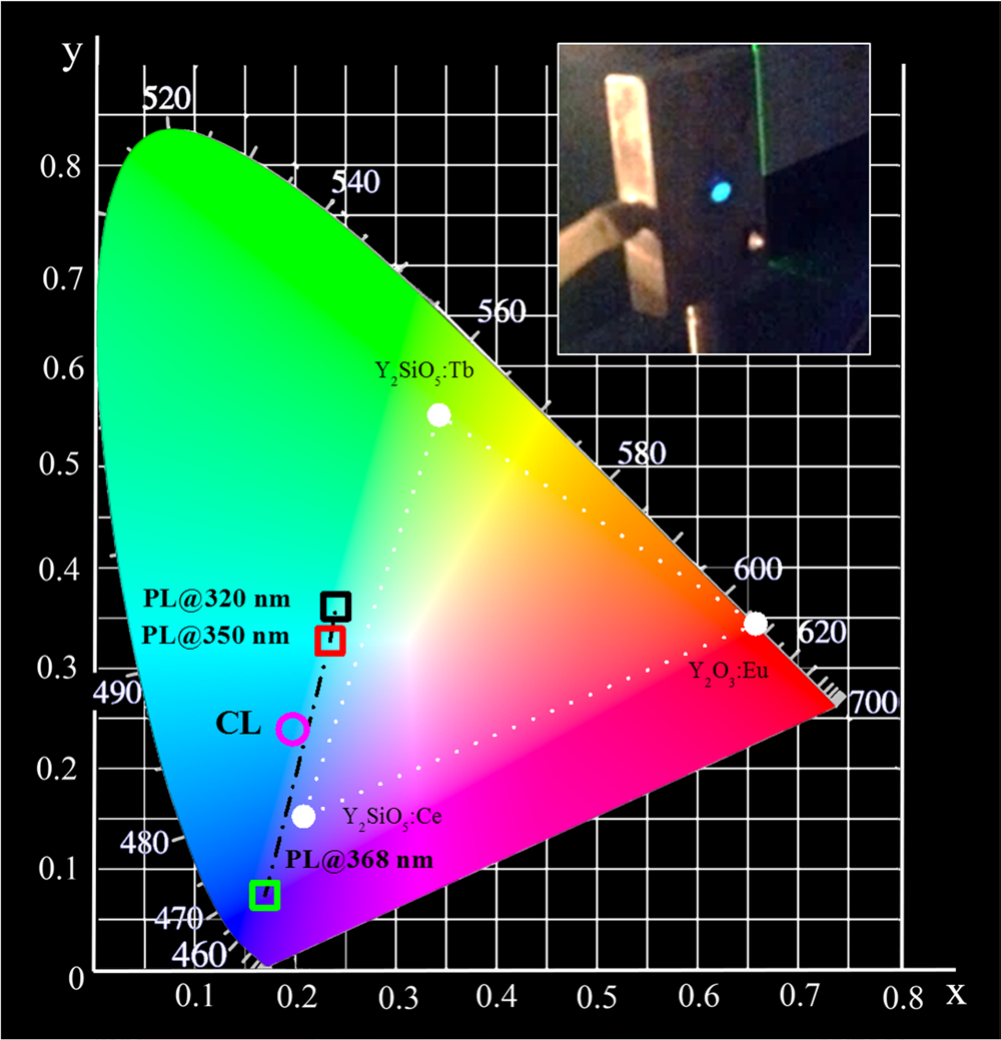
Chromaticity coordinates diagram. The calculated coordinates related to the PL emission from Y_2_O_3_:Bi thin film under optical excitation at different excitation wavelengths from 300 nm to 400 nm are indicated as a dashed-dot line. In particular the coordinates related to 320 nm, 350 nm and 368 nm excitation wavelengths are highlighted as open squared symbols in the diagram. Instead the chromaticity coordinates under 3 keV electron beam excitation are reported as an open circle. The standard triangle for tricolor FED is also reported in the diagram: it consists of the three CIE chromaticity coordinate points of Y_2_O_3_:Eu, Y_2_SiO_5_:Tb, Y_2_SiO_5_:Ce^34,35^. In the insert a photograph of our Bi doped sample under 320 nm excitation.

The PL emission from Bi is visible with the naked eye, as shown in the inset of Fig. 3 through a photograph of the Bi-doped sample under optical pumping at 320 nm excitation wavelength (only green light is observable). Under this excitation condition an internal quantum efficiency (IQE), defined as the ratio between the number of photons created and the number of photons absorbed, has been evaluated as the ratio of effective and radiative lifetime. By measuring the time resolved PL and known the radiative lifetime^24,31^, of Bi^3+^ in the C_2_ site, an IQE value of about 35% is obtained under 325 nm excitation. The obtained IQE value appears comparable or higher than the values reported in literature for powder systems, see for example the 4% efficiency reported for green emission of Mg_2_SnO_4_:Mn^2+^ powders^8^, or the reported value of 35.2% for Eu and Tb codoped silicate thin films^32^. The same estimation is also obtained by a measurement of the external quantum efficiency (EQE) evaluated as the ratio of the emitted power and the incident power by measuring the emitted power through a calibrated spectrometer. The obtained EQE value is equal to 0.05%. By taking into account the low extraction efficiency of the emitted photons from the planar thin film, due to internal reflection losses at the interface host/air, and the very low effective absorbed pumping power by the system ascribed only to the Bi^3+^ ions excitation (^1^S_0_ → ^3^P_1_), we have estimated a similar value of about 35% for IQE.

In order to obtain the true color of the broad emission from the Y_2_O_3_:Bi sample, the color gamut of our light source was evaluated. This is an important index for determining the colors that a display could present, as determined by the location of the Commission International de l’Eclairage (CIE) coordinates of phosphors^33^. Figure 3 reports the calculated chromaticity coordinates diagram for the Y_2_O_3_:Bi with the tricolor gamut triangle used as reference for oxide-based FEDs^34,35^. The values obtained under optical pumping for the excitation wavelengths varying between 300 nm and 400 nm are highlighted with a black dashed-dot line. For example, under the 320 nm and the 368 nm excitation wavelengths, that selectively excite C_2_ or S_6_ sites, coordinates of (0.2403, 0.3594) and of (0.1725, 0.0736) have been obtained, respectively. Therefore, it is possible to tune the PL emission in a continuous way from the green to the violet by just tuning the excitation source, thus suggesting this material suitable as a switchable blue/green emitter for pc-LEDs. Moreover, the chromaticity coordinates associated to the PL emission obtained under 368 nm are very close to the ones of the pure blue (0.1666, 0.0089). It demonstrates higher color purity of our sample than the commercially available blue phosphor such as Y_2_SiO_5_:Ce^35^, reported in the diagram in Fig. 3.

To understand the potentialities of this luminescent thin films in FEDs, the emission under electron beam excitation was further investigated by cathodoluminescence (CL) measurements and compared with the PL properties. The electrons energy was chosen in order to center the electron scattering distribution (as evaluated by Monte Carlo simulations^36,37^), to the Bi^3+^ distribution profile in the film. The penetration depth, *L*, of the electron beam was also evaluated in a first approximation^2^ by the following formula

1 $$L[\dot{{\rm{A}}}]=250\times (\frac{A}{\rho })\times {(\frac{E}{\sqrt{Z}})}^{n},n=\frac{1.2}{1-0.29\times \,\mathrm{lg}\,Z}$$

where A is the atomic or molecular weight of the material (225.78 for the undoped Y_2_O_3_ and 228.34 for Y_2_O_3_:Bi), ρ is the density (5.01 g/cm^3^ for Y_2_O_3_), Z is the atomic number (102.9) and E is the excitation voltage. Due to the fact that low voltage phosphors are currently required by the FEDs technology^2^, the beam acceleration energy was kept fixed at 3 keV. The estimated penetration depth for this energy was about 35 nm and the electron scattering distribution has been simulated to reach 92 nm in depth.

**Figure 4:**
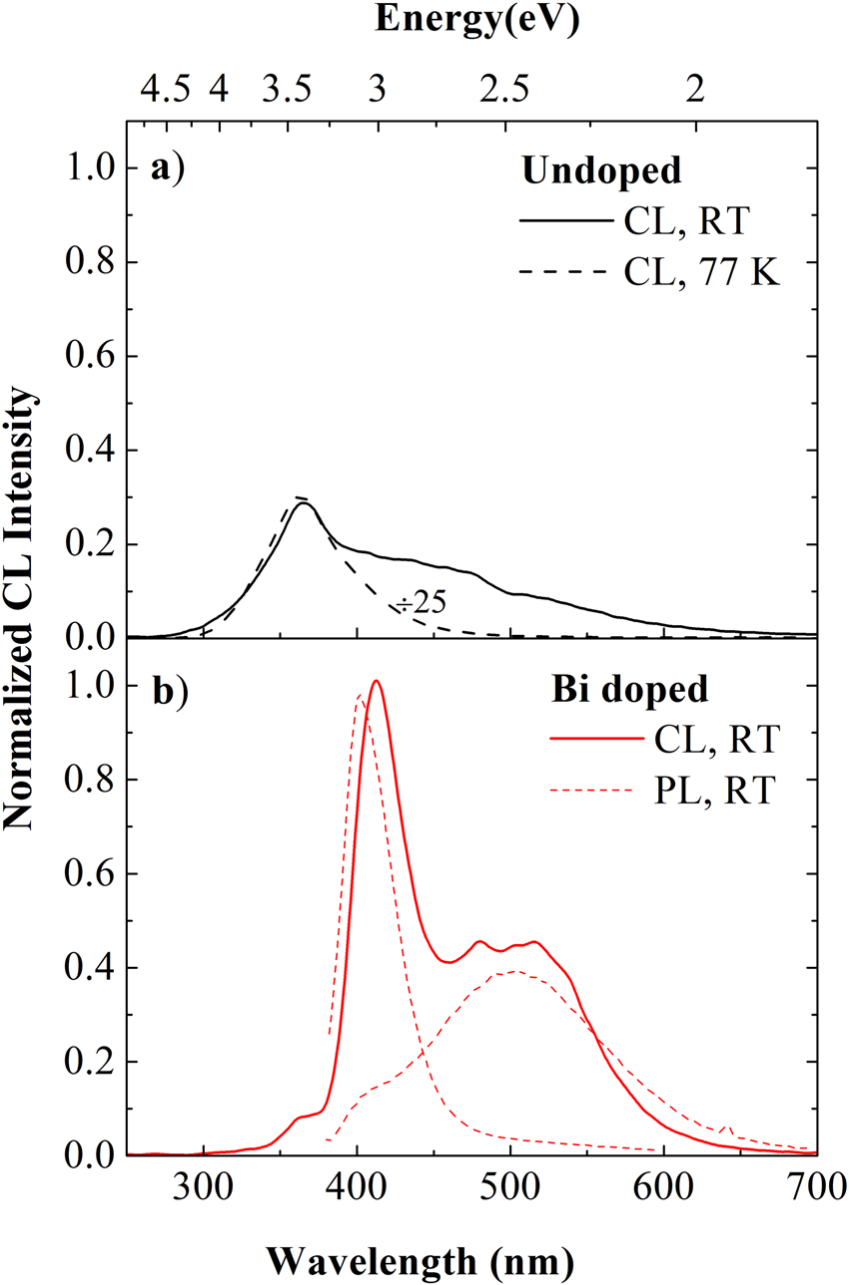
Emission spectra under electron beam excitation. CL spectra (**a**) of undoped Y_2_O_3_ thin film at RT and at 77 K and (**b**) of Bi doped Y_2_O_3_ thin film at RT under electron beam excitation at 3 keV and 5 nA. The intensities reported in the two panels are directly comparable. Figure 4 (**b**) reports also the PL emission spectra from Bi ions in the two sites from the same sample at RT.

Figure 4 shows the comparison between the CL spectra from the undoped and Y_2_O_3_:Bi samples under 3 keV electron beam excitation. Differently from the optical excitation, an emission comes also from the undoped sample, as shown in Fig. 4(a): it is composed by a main peak at 363 nm overlapped with a less intense broad band in the visible range extended down to 600 nm. The nature of the main peak at 363 nm was clarified thanks to the CL measurements at low temperature: its intensity strongly increases, by a factor of 25, by decreasing the temperature up to 77 K without suffering from position shifts, while the visible band, that is kept unchanged, is almost not appreciable. The peculiar behavior of the emission at 363 nm can be ascribed to self-trapped excitons (STE)^38,39^. Indeed, in a variety of wide-bandgap non-semiconducting materials with sufficiently strong exciton–phonon interactions, the self-trapping of free excitons associated to lattice defects can occur. Radiative recombination of STE represents an intrinsic luminescence process, characterized by a broad emission spectrum^40,41^, that occurs when the excitation energies overcome the matrix band gap. The STE excitation condition are thus obtained under electron excitation and not under the investigated optical UV excitation.

Instead concerning the emission band extended down to 600 nm, the lower intensity as well as the co-presence of many not-well resolved bands did not allow us to unambiguously identify their origin. However, a broad emission falling in the visible range has been already observed, as in other inorganic oxides^37,42^, from bulk or powders Y_2_O_3_^38,43^, but the origin was not fully discussed. Only a few works claim that it can be de-convoluted in a broad band at around 428 nm (2.9 eV), related to anion sublattice radiative de-excitation (O^2+^  + h^+^  + e^−^ → O^2−^ + e^−^ → (O^2−^)^∗^ → O^2−^ + *h*ν^43^), and in higher-lying wavelengths bands ascribed to the radiative recombination of the excited donor−acceptor Y^3+^−O^2^− pairs, that contribute with different bands corresponding to various Y−O distance^43^. From the other side, we cannot exclude the further contribution from a few nanometers-thick interfacial silicon oxide layer formed after the annealing treatment, since it is well known that also SiO_x_ layer exploits radiative transitions coming from defects in the same wavelength range^44^, such as for instance twofold coordinated silicon centers (at about 468 nm), carbon contaminants (at about 520 nm) and the Non Bridging Oxygen Hole Center (at 633 nm).

When the Bi-doped sample is excited under the same conditions at RT, a totally different CL spectrum is observed as reported in Fig. 4(b). The yttrium oxide STE contribution appears very low and the spectrum is mainly composed by a sharp emission peaked at 412 nm and by a broader contribution at around 500 nm. These two features well match the Bi^3+^ emission bands already observed under optical pumping (reported as dashed lines); in particular the sharp peak corresponds to the Bi^3+^ emission in the S_6_ lattice sites, while the broad visible band can be decomposed by a broad band peaked at about 512 nm corresponding to the Bi^3+^ emission in the C_2_ sites and a weaker contribution related to the radiative defect centers from the host and the interfacial silica layer, already observed in the undoped sample. Thus, under electrical pumping we observe luminescence from Bi in both lattice sites. The slight peaks shift (<10 nm) in the Bi^3+^ CL with respect to the PL could be ascribed to the presence of charge effects and local oxidation mechanisms^45–47^ that can lead to a shift of the ^3^P_1_ shell, since the energy position is strongly dependent on the ion environment. This mechanism is possible only under electron beam excitation, due to the much higher energy involved, 3 keV, with respect to the 3.8 eV of the exciting photons.

**Figure 5:**
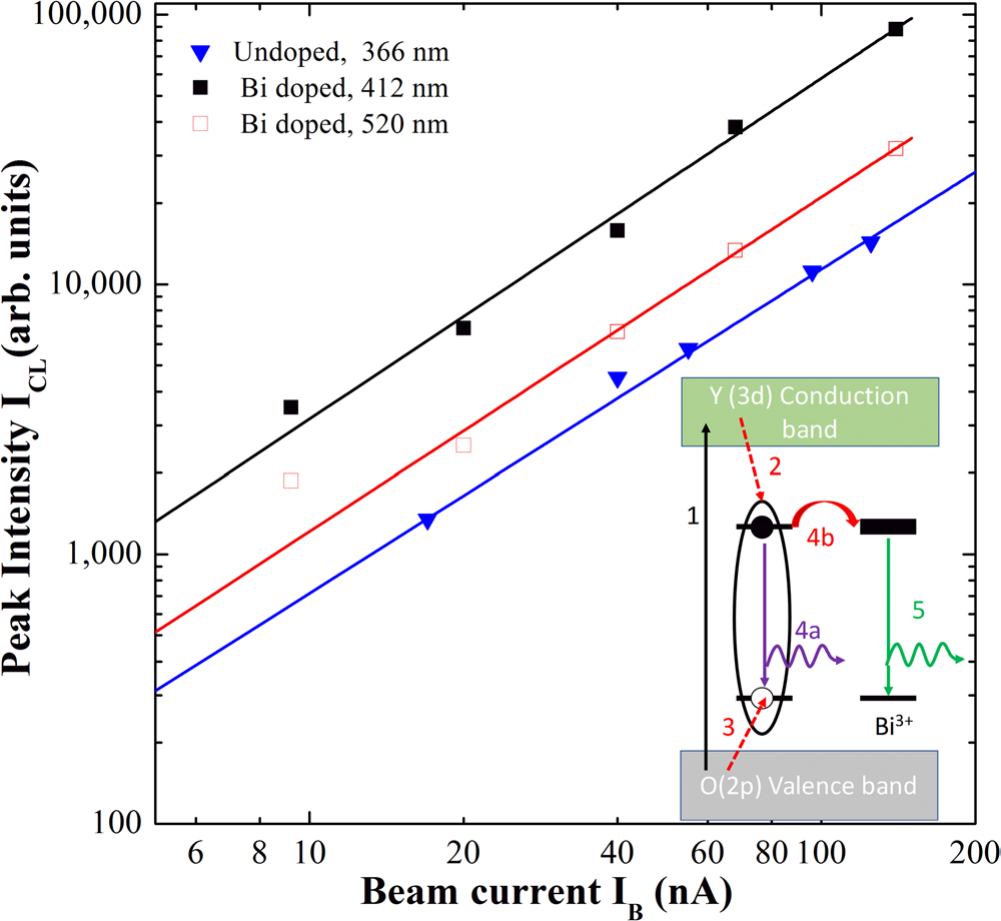
Cathodoluminescence – beam current relationship for undoped and doped samples. RT CL peak intensity versus beam current, recorded at 366 nm from undoped Y_2_O_3_ and at 412 nm and 520 nm from the doped film at RT under electron beam irradiation at 3 keV. The fits are also reported as continuous lines. A schematic of the proposed excitation mechanisms is reported as inset, starting from the above-gap excitation through highly energetic electrons (1), creation of the STE (2–3), STE emission (4a) or ET in presence of Bi (4b) and finally Bi emission (5).

The CL emission was verified to be stable also after several hours of excitation conditions. To go further in testing the potentialities of Y_2_O_3_:Bi as a possible visible emitter in FEDs, we have investigated the dependence of the CL emission on the electron beam current (I_B_) in the range between 9 nA and 110 nA. Both for undoped and doped samples the emission shapes appear unchanged in all the investigated range. Therefore in Fig. 5 we have plotted the peak CL intensity at 366 nm for the undoped, corresponding to the STE emission, and at 412 nm and at 520 nm for the Bi-doped samples, corresponding respectively to the S_6_ and C_2_ site, as a function of the beam current.

Figure 5 shows that the CL intensity increases with the electron beam current for undoped and Bi doped sample. The dependence of CL intensity (I_CL_) on electron current density (j) can be described by a power law I_CL_ ∝ j^m^ ^42^, where the value *m* can give some information concerning the process involved in the excitation and de-excitation mechanisms as well as the nature of the involved emitting centers. By keeping constant the excited area during the CL measurements, it was possible to study the I_CL_ dependence versus the beam current I_B_. We performed a fit of the experimental data in the log−log scale and the *m* values were extracted for both samples. The fits are reported in Fig. 5 as continuous lines, as well as the obtained *m* values. In details, a value of 1.20 ± 0.02 was obtained for the undoped sample and of 1.24 ± 0.02 and 1.26 ± 0.02 for the two emission peaks from the doped sample. The superlinear CL dependence on the current density has been demonstrated to be typical of recombination channels related to free and bound excitons^48^. Therefore, the obtained *m* value of 1.20 for the undoped Y_2_O_3_ further supports the occurrence of STE emission even at RT. Another interesting point is that under electronic excitation similar values are obtained also for the Bi emission at 412 nm and 520 nm in the doped sample, differently from the optical pumping where a simple linear dependence has been obtained (not shown). This behavior suggests also in this case the involvement of STE in the Bi excitation mechanisms. This claim is also supported by the strong reduction of the STE peak intensity observed when Bi is introduced. Indeed, in this respect we can suppose that an energy transfer (ET) process from the yttrium oxide STE to the Bi^3+^ ions in both sites occurs. This ET could be made possible by the large spectral overlap between the exciton emission band peaked at 363 nm (see Fig. 4) and the broad Bi excitation bands peaked at 330 nm and 368 nm for the C_2_ and S_6_ sites, respectively (see in Fig. 2(a) and (b)), as already suggested for Eu:Y_2_O_3_^39^ and for RE-doped semiconductors^41^.

An evaluation of the cathodoluminescence efficiency was carried out following the methods by Shea and Walko^49^. This method allows an evaluation of the CL efficiency, considering the material properties and the electron excitation parameters, and it is comparable to the EQE. The CL efficiency with a beam current of 20 nA is 0.06% and 0.004% in the case of the Bi doped and the undoped material, respectively. Eventually, the chromaticity coordinates of the Y_2_O_3_:Bi light source under electron beam irradiation have been calculated and reported by a circle in Fig. 3 in comparison with the ones obtained under optical pumping. Color coordinates of (0.1972, 0.2377), corresponding to a light blue emission, have been obtained. These coordinates are outside those of the traditional FED tricolor gamut^34,35^, and they could be interesting to enlarge the FEDs color gamut when combined with RGB tricolor phosphors.

## Conclusions

In this paper, we have discussed the emission properties of Bi-doped yttrium oxide thin films on c-Si. In particular, we have demonstrated that under optical pumping Y_2_O_3_:Bi exhibits a stable and strong visible luminescence at RT which can be tuned from violet to green by changing the excitation wavelength, due to the involvement of Bi ions in the two available lattice sites. Internal quantum efficiency of about 35% has been evaluated. Those data suggest the suitability of Bi-doped Y_2_O_3_ thin films as luminescent material for pc-LEDs, acting as a tunable violet-green light source or a pure blue source. Moreover, under electronic beam a light blue emission is obtained, being the convolution of the green and blue light from Bi in the two lattice sites, permitting to enrich the standard color gamut of FEDs. Bi excitation mechanisms have been explained as an energy transfer process between the Y_2_O_3_ STE and Bi ions, as evidenced by the superlinear CL behavior with the beam current. These results, together with the high emission efficiency under low accelerating voltages (3 keV) and the excellent stability, suggest the suitability of Bi-doped Y_2_O_3_ thin films as a pure light blue source in FEDs, and opening new perspectives for the realization of CMOS-compatible light sources operating at room temperature.

## Materials and Methods

### Materials synthesis

Undoped Y_2_O_3_ thin films were deposited on 5″ c-Si wafers by radio-frequency magnetron sputtering. The substrate was heated at 400 °C during the synthesis process and the power applied to the Y_2_O_3_ target was fixed at 500 W. We obtained a polycrystalline stoichiometric yttrium oxide film, 120 nm thick, as verified by Rutherford Back-scattering Spectrometry (RBS) and X-ray diffraction analyses (XRD). More details can be found in ref.^20^. Bi ions were then introduced by ion implantation at 270 keV, that results in a Bi profile spread over a thickness of about 80 nm and peaked at about 35 nm below the surface, as measured by RBS. The Bi dose was kept fixed at 2 × 10^15^ at/cm^3^ and the respective average Bi concentration was evaluated to be 3 × 10^20^ Bi/cm^3^. The undoped and as-implanted samples were then annealed at 800 °C for 30 min in oxygen atmosphere to recover eventual oxygen deficiencies and remove defects left over by the implantation process. The stoichiometry and dopant distribution were unchanged after annealing treatment. Also the polycrystalline structure was preserved. In particular, the body centered cubic crystalline structure of Y_2_O_3_ was identified by XRD^30^, that excludes the formation of crystalline Bi_2_O_3_, by demonstrating the good dispersion of Bi in the Y_2_O_3_ lattice in substitutional Y positions.

### Optical Characterization

The optical properties of all the annealed undoped Y_2_O_3_ and Y_2_O_3_:Bi thin films have been analyzed by photoluminescence (PL) and photoluminescence excitation (PLE) spectroscopy at room temperature (RT), performed by a Horiba Fluorolog spectrofluorimeter equipped with a 400 W Xe lamp. The excitation wavelength was varied between 300 nm and 550 nm with a scan step of 1 nm, while the luminescence was detected by a Hamamatsu Photomultiplier.

Cathodoluminescence (CL) properties have been investigated using a commercial Oxford CL system, fitted onto a Cambridge S360 standard tungsten gun scanning electron microscope. The CL system is equipped with a 1800 line/mm grating and a multialkali photomultiplier sensitive in the range 350–830 nm. The spectroscopic CL analyses are carried out with an accelerating voltage of 3 kV at room temperature and at low temperature (liquid nitrogen, 77 K). The spectral resolution is 5 nm.
